# Computer-Assisted Algorithm for Quantification of Fibrosis by Native Cardiac CT: A Pilot Study

**DOI:** 10.3390/jcm13164807

**Published:** 2024-08-15

**Authors:** Diana Gonciar, Alexandru-George Berciu, Eva-Henrietta Dulf, Rares Ilie Orzan, Teodora Mocan, Alex Ede Danku, Noemi Lorenzovici, Lucia Agoston-Coldea

**Affiliations:** 1 2nd Department of Internal Medicine, Iuliu Hațieganu University of Medicine and Pharmacy, 400012 Cluj-Napoca, Romania; dianagonciar@gmail.com (D.G.); orzanrares@gmail.com (R.I.O.); luciacoldea@yahoo.com (L.A.-C.); 2 Automation Department, Faculty of Automation and Computer Science, Energy Transition Research Center, Technical University of Cluj-Napoca, 400114 Cluj-Napoca, Romania; eva.dulf@aut.utcluj.ro (E.-H.D.); alex.danku@aut.utcluj.ro (A.E.D.); noemi.lorenzovici@gmail.com (N.L.); 3 Physiological Controls Research Center, University Research and Innovation Center, Obuda University, 1034 Budapest, Hungary; 4 Physiology Department, “Iuliu Hațieganu” University of Medicine and Pharmacy, 400012 Cluj-Napoca, Romania; teodora_mocan@yahoo.com; 5 Department of Nanomedicine, Regional Institute of Gastroenterology and Hepatology, 400158 Cluj-Napoca, Romania

**Keywords:** artificial intelligence, cardiac imaging, HER2+ Breast cancer, myocardial fibrosis

## Abstract

**Background/Objectives:** Recent advances in artificial intelligence, particularly in cardiac imaging, can potentially enhance patients’ diagnosis and prognosis and identify novel imaging markers. We propose an automated, computer-aided algorithm utilizing native cardiac computed tomography (CT) imaging to identify myocardial fibrosis. This study aims to evaluate its performance compared to CMR markers of fibrosis in a cohort of patients diagnosed with breast cancer. **Methods:** The study included patients diagnosed with early HER2+ breast cancer, who presented LV dysfunction (LVEF < 50%) and myocardial fibrosis detected on CMR at the time of diagnosis. The patients were also evaluated by cardiac CT, and the extracted images were processed for the implementation of the automatic, computer-assisted algorithm, which marked as fibrosis every pixel that fell within the range of 60–90 HU. The percentage of pixels with fibrosis was subsequently compared with CMR parameters. **Results:** A total of eight patients (n = 8) were included in the study. High positive correlations between the algorithm’s result and the ECV fraction (r = 0.59, *p* = 0.126) and native T1 (r = 0.6, *p* = 0.112) were observed, and a very high positive correlation with LGE of the LV(g) and the LV-LGE/LV mass percentage (r = 0.77, *p* = 0.025; r = 0.81, *p* = 0.015). A very high negative correlation was found with GLS (r = −0.77, *p* = 0.026). The algorithm presented an intraclass correlation coefficient of 1 (95% CI 0.99–1), *p* < 0.001. **Conclusions:** The present pilot study proposes a novel promising imaging marker for myocardial fibrosis, generated by an automatic algorithm based on native cardiac CT images.

## 1. Introduction

Myocardial fibrosis represents the excessive accumulation of collagen in the extracellular space and is classified into two main types: replacement and diffuse. Due to the heart’s limited regenerative capacity, extensive cardiomyocyte necrosis triggers a significant tissue repair process, resulting in the formation of a fibrous scar (replacement fibrosis), which is essential to prevent the rupture of the ischemic myocardium [1]. In contrast, diffuse fibrosis arises from various physiological factors (such as aging) and pathological conditions (including volume overload, pressure overload, inflammatory factors, and cardiomyopathies) [2]. It affects the interstitium or the perivascular area, leading to the onset or aggravation of heart failure (HF) due to impaired microanatomy and function [3]. Identifying and quantifying diffuse fibrosis is critical, not only because it holds prognostic value but also because it is potentially reversible with appropriate management [3].

Cardiotoxicity among patients with breast cancer and human epidermal growth factor receptor 2 (HER2) gene amplification, primarily manifested as asymptomatic left ventricular (LV) dysfunction, is a well-known complication of the standard treatment. This condition is induced by both anthracyclines [4] and targeted therapy with monoclonal antibodies (trastuzumab) [5]. The cumulative incidence of HF and cardiomyopathy over three years increased from 26.7% to 28.2% with the simultaneous administration of these two drugs [6]. Although echocardiography is used for the initial assessment, cardiac magnetic resonance (CMR) imaging provides superior sensitivity and reproducibility, allowing for the identification of myocardial fibrosis that precedes clinical cardiac dysfunction [7].

CMR is central to the multimodal imaging workup, providing anatomical and functional evaluation and tissue characterization. Late gadolinium enhancement (LGE) is the standard non-invasive tool for evaluating replacement fibrosis [8], resulting from extracellular space expansion or insufficient drainage [9]. However, LGE is not suitable for evaluating diffuse fibrosis due to poor spatial resolution and the potential absence of unaffected myocardium [3]. Native CMR T1 mapping effectively highlights interstitial fibrosis through prolonged T1 relaxation times, correlating well with histology assessment [10]. This technique is particularly useful in patients with chronic renal disease, where gadolinium-based contrast agents cannot be used [8], and it offers reduced time and costs [10].

Myocardial strain represents the percentage change in the size of a myocardial segment compared to its initial size [11]. Global longitudinal strain (GLS) is a marker of subclinical myocardial dysfunction, observed to change in patients undergoing anti-HER2 therapy [12] and those receiving anthracyclines [13]. In vivo studies have shown that GLS correlates with the collagen volume fraction [14] and has a predictive role in the mortality of patients with cardiomyopathy [15].

Despite its advantages, the clinical use of CMR is limited by its reduced availability, high costs, and contraindications [16]. Consequently, computed tomography (CT) is gaining attention as an alternative for evaluating myocardial function and tissue characterization, in addition to its established role in assessing coronary anatomy. Several studies have reported a good correlation between the extracellular volume (ECV) fraction determined by CT and CMR [17,18]. However, a significant limitation is the increased dose of contrast medium required [16], and to our knowledge, no methods have been reported that emphasize myocardial fibrosis on native cardiac CT (CCT).

In recent years, automated diagnostic methods based on machine learning have gained popularity and have been successfully used in areas such as detection of colorectal polyps [19], celiac disease [20], or even cancer [21].

The present paper proposes a novel computer-aided diagnostic method for identifying and quantifying cardiac fibrosis on native CCT images. The study aimed to test its performance on a retrospective cohort of patients diagnosed with HER2-positive breast cancer.

## 2. Materials and Methods

### 2.1. Study Population

An observational study was conducted involving patients with HER2-positive early breast cancer and a diagnosis of LV dysfunction associated with chemotherapy. The study was conducted at the 2nd Medical Clinic of the County Emergency Hospital Cluj-Napoca and the Hiperdia Diagnostic Imaging Centre Cluj-Napoca between October 2021 and October 2022, in accordance with the principles of the Declaration of Helsinki. The study protocol was approved by the Ethics Committee of the “Iuliu Haţieganu” University of Medicine and Pharmacy Cluj-Napoca (approval number 257/30.06.2021). Eligible patients were informed about the study protocol and provided written informed consent.

Inclusion criteria: Patients were included if they had LV dysfunction (LV ejection fraction (LVEF) ≤ 50%) and the presence of myocardial fibrosis on CMR, were over 18 years old, and were recently diagnosed with early-stage breast cancer (stage I to IIIA) HER2 positive, and treated with anthracycline-based chemotherapy and trastuzumab.

Exclusion criteria: Patients were excluded if they: (1) refused to participate in the study after being informed; (2) had poor acoustic windows; (3) had contraindications for CMR examination (e.g., cardiac stimulators, implantable cardioverter defibrillators, cardiac resynchronization therapy devices, metal implants or impacted metal objects); (4) had a life expectancy of less than one year due to the progression of myocardial disease or other causes; (5) had a history of significant cardiovascular diseases (e.g., cardiac malformations, myocardial infarction, valvulopathies, myocarditis, cardiomyopathies; atrial fibrillation); (6) had other conditions that could contribute to myocardial fibrosis and influence the results (e.g., history of cardiac surgery); (7) had severe renal insufficiency or severe allergy to contrast agents; (8) had a history of radio- and/or chemotherapy for a prior malignancy; or (9) were pregnant or breastfeeding.

Demographic data collected for all patients included age, sex, height, weight, medical history, cardiovascular symptoms (chest pain, dyspnea, syncope, palpitations), and current medication. Additionally, a 12-lead electrocardiogram (ECG) was recorded. Twenty-four-hour ECG Holter monitoring and transthoracic echocardiography were also performed. CCT was conducted within the first 10 days after CMR.

### 2.2. CMR Protocol and Image Analysis

CMR was performed using a 1.5 Tesla imaging system (Magnetom Altea, Siemens Medical Systems Solutions, Erlanger, Germany), ECG-gated, and conducted during complete apnea, following a standard protocol in accordance with international guidelines [22].

The function and the mass of the LV were evaluated in longitudinal and short axes, from the base to the apex, using steady-state free precession (SSFP) sequences. Standardized acquisition parameters ensured adequate blood-cavitary wall discrimination: repetition time (RT) = 3.6 ms, eco-time (ET) = 1.8 ms, flip angle = 60°, section thickness = 6 mm, field of view = 360 mm, image matrix = 192 × 192 pixels, voxel size = 1.9 × 1.9 × 6 mm, and temporal resolution = 25–40 ms reconstructed to 25 phases of the cardiac cycle [23].

The endocardial and epicardial regions were demarcated in telesystole and telediastole using Syngo.via software (version VB20A_HF08, Argus, Siemens Medical Solutions, Oakville, ON, Canada).

A modified inversion recovery Look-Locker sequence was used for T1 mapping, focusing on one mid-ventricular short-axis slice. T1 relaxation time was determined by establishing a conservative region of interest in the myocardial septum [24]. The limit for native T1 times was considered to be 950 ± 21 ms [25].

Images for T1 measurements were obtained before and after an intravenous infusion of 0.2 mmol/kg gadobutrol (Gadovist, Bayer Schering Pharma AG, Berlin, Germany) was administered. The ECV was calculated using the formula $ECV=\left(1-haematocrit\right)\times [(\frac{1}{pT1m}-\frac{1}{nT1m})/(\frac{1}{pT1b}-\frac{1}{nT1b})]$, where nT1 represents native T1, pT1 is post-contrast T1, m refers to myocardium, and b refers to blood pool, with reference values of 25 ± 4% [25].

### 2.3. CCT Protocol and Image Analysis

CCT images were acquired using a second-generation single-source CT scanner (Siemens SOMATOM Definition Edge, Siemens Healthcare, Erlangen, Germany) with a 0.6 mm slice thickness and 1.5 mm reconstruction interval [26]. The X-ray tube voltage ranged from 70 to 140 kV, the tube current was between 500 and 650 mAs per rotation, and the section thickness was 3 mm.

Pre-contrast calcium scoring was assessed semi-automatically using Syngo Calcium Scoring software (CT VC28, Siemens AG, Erlangen, Germany). The Agatston score was calculated, defining a lesion as at least four contiguous pixels, an area greater than 1 mm^2^, and a density of ≥130 Hounsfield Units (HU) [27].

Coronary images were acquired covering the entire cardiac cycle following an intravenous injection of 1.5 mL/kg of contrast medium, divided into two boluses: 80–100 mL of non-ionic contrast medium (Omnipaque 350 mgI/mL, GE Healthcare, Princeton, NJ, USA) administered with a dual-head power injector (SCT 210; Medrad, Warrendale, PA, USA), followed by 50–80 mL of saline solution. Data acquisition started 5 s after reaching the signal attenuation threshold. For patients with heart rates below 65 bpm, a prospective ECG-gated protocol was implemented, scanning 70–80% of the R-R interval. A postcontrast cardiac CT scan was performed 20 min later with parameters identical to those used for the pre-contrast calcium score scan.

All CCT and CMR interpretations were performed by two Level III-trained experts who were blinded to the clinical data.

### 2.4. Algorithm Protocol

A novel algorithm was developed by the researchers from the ADAPTED Research Group, part of the Energy Transition Research Center within the Technical University of Cluj-Napoca, Romania. The initial phase involves enumerating the patients within the database and assigning unique identification numbers to each, ensuring a comprehensive index essential for subsequent image analysis and processing.

The number of CT images available for each patient is identified, cataloging the volume of images per patient to provide a clear understanding of the data scope for each individual. The original images are read in DICOM format, a standardized format in medical imaging that contains the necessary information for further processing and analysis.

The application of the fibrosis detection algorithm to the DICOM images involves several sub-steps. The DICOM images, formatted as a 3D numerical matrix, are converted into a matrix that includes HU data, which will be utilized in the fibrosis detection process. Heart contour detection is performed using the numerical matrix. This begins with determining a customized set of starting parameters for each image through a series of manual tests, defining the area of interest where the Snake algorithm will search for the desired contour. The Snake algorithm, implemented using Python version 3.11.7 and the scikit-image library version 0.24.0 (specifically the active_contour function), is configured with the following parameters: a. Gaussian filter with a sigma of 3 and preserve_range set to False; b. Initial contour from the previous step; c. Alpha: 0.15, Beta: 5, Gamma: 0.001; d. 10 iterations. The initial contour is represented as a red dashed line, and the detected contour is depicted as a solid blue line. The region of interest (ROI) is defined as the area between the minimum and maximum coordinates where the contour detected by the Snake algorithm evolves, thereby extracting the heart region as a rectangle from the original image.

Using the detected ROI, the fibrosis detection algorithm is applied at the pixel level. A copy of the image showing the heart contour is created, and the ROI is traversed pixel by pixel. Fibrosis is identified if a pixel’s value falls within the range of 60–90 HU, an interval established through repeated comparative measurements between normal and pathological myocardium. Pixels identified as having fibrosis are marked with a different color in the new image created from the initially identified ROI.

### 2.5. Statistics

Data distribution was assessed using the Kolmogorov–Smirnov test. Results are presented as mean ± standard deviation (SD) or 95% confidence interval (CI) if normally distributed, as median (25–75% interquartile range [IQR]) if non-normally distributed, or as percentages. Correlation coefficients (r) were interpreted as follows: >0.7 as very high correlation, 0.5–0.7 as high correlation, 0.3–0.5 as medium correlation, 0.1–0.3 as low correlation, and <0.1 no correlation. Differences between CMR and CT groups were assessed using the paired Student *t*-test.

To evaluate the reliability of the proposed algorithm, the intraclass correlation coefficient (ICC) was determined. ICC values were interpreted as follows: <0.50 indicated poor reliability, 0.50–0.75 suggest moderate reliability, 0.75–0.90 indicated good reliability, and >0.90 indicated excellent reliability [28]. Repeatability was assessed using the coefficient of variance, determined by the root mean square method.

A *p*-value < 0.05 was considered statistically significant. All statistical analyses were performed using DATAtab: Online Statistics Calculator (DATAtab e.U. Graz, Austria. URL https://datatab.net) and MedCalc Statistical Software version 22.032 (MedCalc Software Ltd., Ostend, Belgium; https://www.medcalc.org; 2020).

## 3. Results

**Figure 1:**
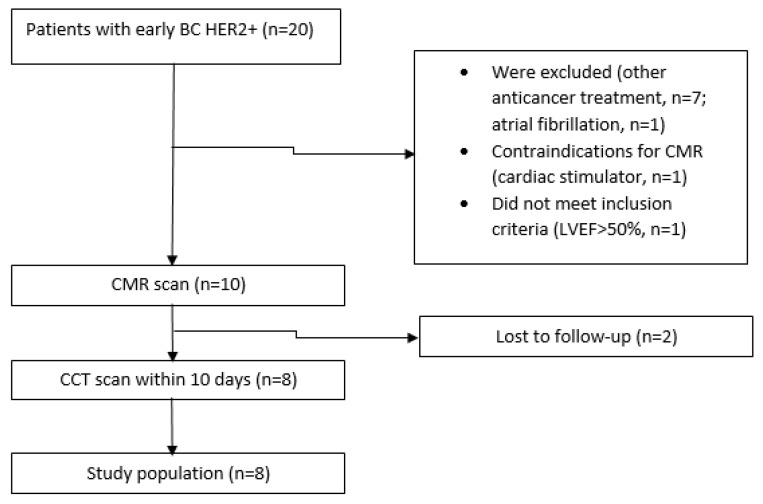
Study population was determined after applying inclusion and exclusion criteria (BC: breast cancer; HER2: human epidermal growth factor receptor 2; LVEF: left ventricle ejection fraction; CMR: cardiac magnetic resonance; CCT: cardiac computer tomography).

A total of eight female patients were included in the study (Figure 1). The mean age of the patients was 54.88 ± 12.05, with an age range of 36–70 years.

Dyspnea was the most prevalent symptom, observed in 75% of the study population. The clinical characteristics of the population are summarized in Table 1.

The parameters determined by CMR and CCT are presented in Table 2. At the moment of diagnosis, the mean LVEF was 46.3% ± 12.05 by CMR and 48.8% ± 1.1 by CCT, with 87.2% of the patients presenting LGE, predominantly in the septal area (3/8).

**Figure 2:**
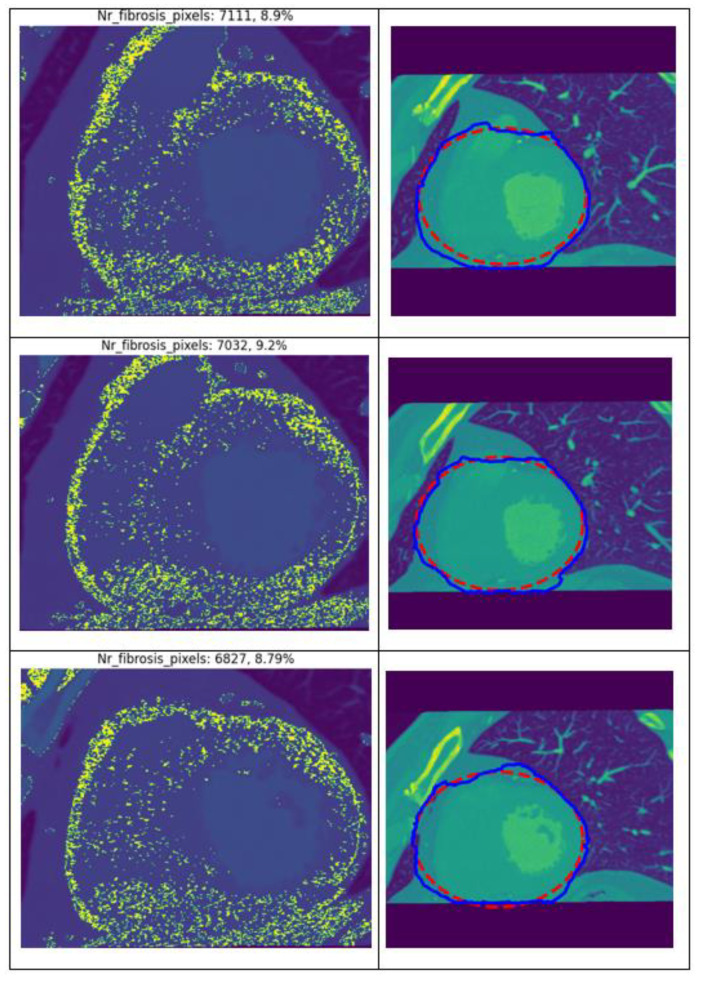
Results generated by the algorithm when using different CT images of the same patient.

The results obtained from applying the developed algorithm to the CT images of patient 1 are presented in Figure 2. The left column illustrates the regions automatically identified by the algorithm as containing fibrotic tissue, indicated by the pixel value, measured in HU, within the range of 60–90 HU. The second column displays the heart region detection using the Snake algorithm applied to the entire CT image. The starting region is marked in red, while the final region, determined after completing the specific iterations, is outlined in blue. Notably, the algorithm successfully identifies both the heart region and areas of fibrosis across different images of the same patient, demonstrating its consistency and reliability when applied to native CT images.

In this situation, the ROI, and also the returned percentages of fibrosis, will be slightly different (Table 3). The coefficient of variance in this case ranged between 1.93% and 15.56%.

**Figure 3:**
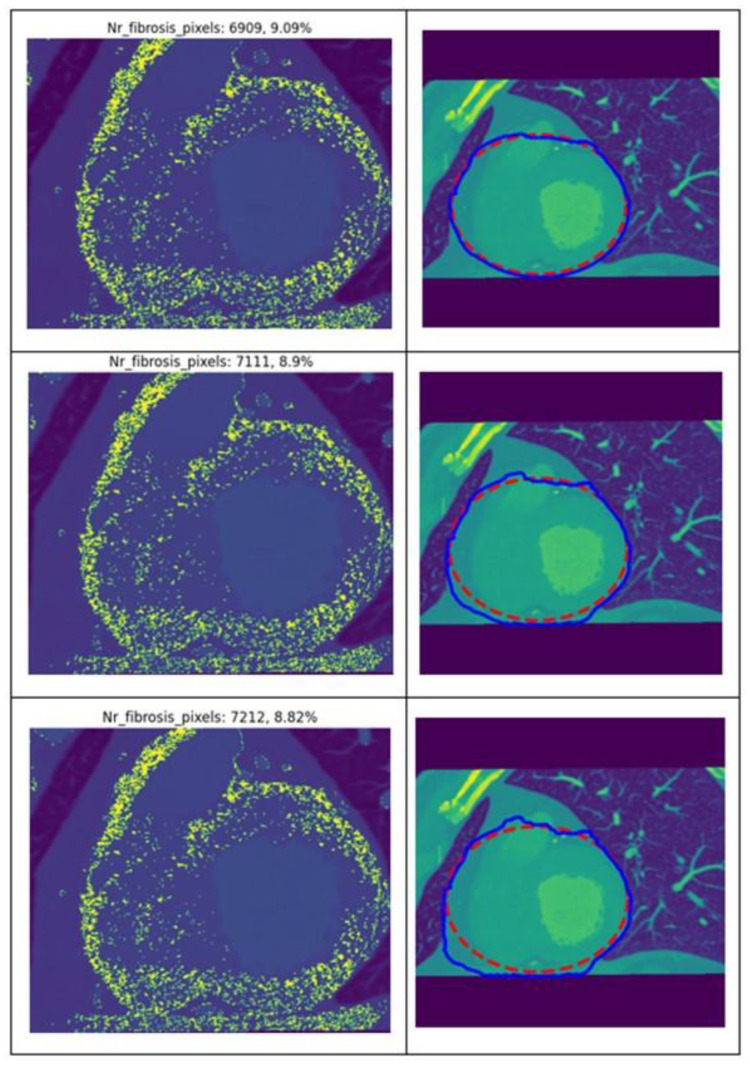
Results generated by the algorithm when using same CT image of patient 1 with different values for the number of iterations of the Snake algorithm.

To evaluate the robustness of the algorithm, the effect was analyzed of increasing the number of iterations by 10 across three successive runs of the Snake algorithm for heart region detection and ROI identification. The initial value was determined through manual testing, and for iterations 2 and 3, the increment was held constant. The results obtained from this analysis are presented in Figure 3, focusing on the first patient and their first CT.

After running the algorithm three times, on the same selected ROI, by the same observer (Table 4), an intraclass correlation coefficient was found of 1 (95% CI 0.99–1), *p* < 0.001. The coefficient of variance ranged between 0.08% and 5.2%.

**Figure 4:**
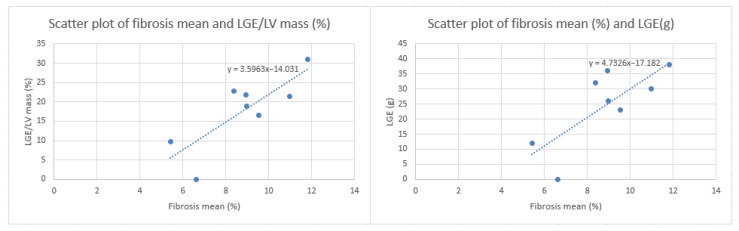
Scatter plot of fibrosis mean (%) compared to LGE/LV mass (%) and LGE (g).

**Figure 5:**
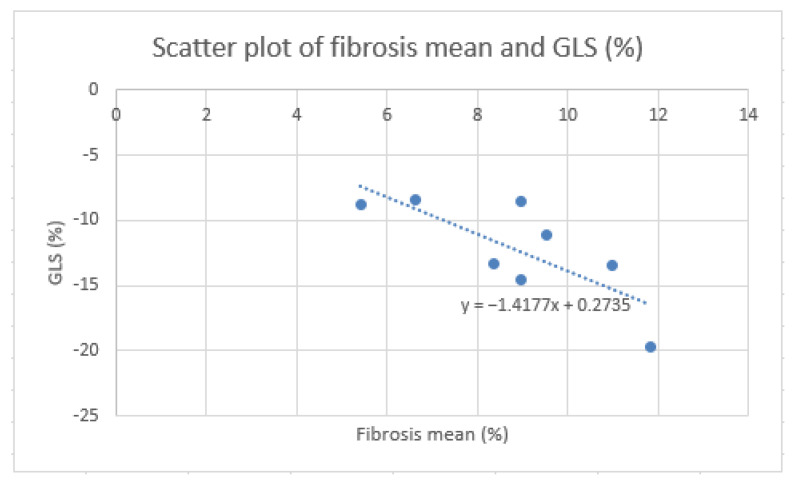
Scatter plot of fibrosis mean (%) compared to GLS (%).

For further evaluation, the mean of three fibrosis percentage determinations was calculated for each patient across three different CT images (Table 3). Pearson correlation analysis indicated a high positive correlation between the fibrosis percentage determined by the algorithm and ECV fraction (r = 0.59, *p* = 0.126), as well as native T1 (r = 0.6, *p* = 0.112). A very high positive correlation was reported between the algorithm’s results and the LGE of the LV(g) and the LV-LGE/LV mass percentage (r = 0.77, *p* = 0.025; r = 0.81, *p* = 0.015) (Figure 4). Additionally, the analysis revealed a very high negative correlation between the GLS percentage and the fibrosis percentage determined by the algorithm (r = −0.77, *p* = 0.026) (Figure 5), which was also observed between GLS percentage and ECV fraction (r = −0.82, *p* = 0.014). The correlation between NT-proBNP levels and the fibrosis percentage, ECV fraction, native T1, LGE(g), or LV-LGE/LV mass percentage was not statistically significant (*p* = 0.697, *p* = 0.716, *p* = 0.911, *p* = 0.744, *p* = 0.842).

## 4. Discussions

In the present study, patients with LV dysfunction, defined by a reduction in LVEF, were included. The observed LVEF values ranged from 39.2% to 50%, consistent with a HF with mid-interval LVEF [29]. Data from the literature indicate that LVEF can change as early as two weeks after the initiation of chemotherapy [30], with possible mechanisms including increased vascular resistance or aortic stiffening secondary to the antitumor treatment [31].

In the context of chemotherapy-induced cardiotoxicity, myocardial strain emerges as the earliest altered parameter, often preceding changes in LVEF [32]. Longitudinal strain is represented by negative values, indicating longitudinal shortening of the heart from base to apex [33]. In our study, the GLS was −12.25 ± 3.87%, which is above the pathological threshold of −17%. GLS is particularly sensitive to lesions in the subepicardial and subendocardial regions and has been correlated with the extent of LGE, suggesting it may serve as an indirect parameter for fibrosis [33]. Consistent with our findings, a high statistically significant negative correlation was observed between GLS and LGE, as well as GLS and LGE/LV mass (Pearson correlation coefficients r = −0.79, *p* = 0.02; r = −0.83, *p* = 0.01).

LGE patterns can be broadly classified into ischemic and non-ischemic types [34]. However, in the context of anticancer therapy, it has been noted that the pattern and location of LGE do not conform to a single model; any pattern can manifest in any location [35]. Our study similarly did not reveal a clear predilection for a specific LGE model.

In patients treated with anthracyclines, T1 relaxation time can change relatively early, even before reductions in LVEF are observed [4,36], and increased ECV fraction is directly correlated with anthracycline administration [37]. Changes in these parameters among chemotherapy-treated patients occur independently of tumor presence or other cardiovascular risk factors [38]. In our study, both elevated T1 (1114 ± 51 ms) and ECV (31.2 ± 3.1%) were observed, indicating the presence of diffuse fibrosis, although we did not achieve a significant correlation with decreased LVEF (*p* = 0.527). The ECV fraction showed a statistically significant correlation with GLS, supporting previous reports of an association between myocardial strain and diffuse fibrosis in patients with lower LVEF [39].

The automatic algorithm proposed in this study demonstrated reliability, with excellent correlation in repeated measurements performed by the same observer. The coefficient of variation analysis revealed an average percentage of 1.52 ± 1.65%, indicative of high precision. This algorithm, utilizing native CT images, effectively highlighted myocardial fibrosis in HER2-positive breast cancer patients, exhibiting a very high and statistically significant correlation with the amount of LGE, along with a strong correlation with ESV and T1 relaxation time. Notably, the percentage of fibrosis generated by the algorithm correlates with both focal and diffuse fibrosis, and the statistically significant correlation with GLS may be attributed to underlying fibrosis influencing myocardial strain.

In recent years, determining ECV via CT has gained considerable attention, yet it requires a specialized protocol involving delayed-phase acquisitions, which are limited by the use of contrast agents and associated risks, as well as increased radiation exposure [40]. The implementation of a deep-learning algorithm has the potential to partially improve these shortcomings, being able to highlight fibrosis by evaluating early-enhanced CCT sequences [41]. Overall, artificial intelligence solutions have increasingly supported clinicians and patients, enabling automatic segmentation, lesion identification, image reconstruction, and assessment of coronary stenosis in CT images [42].

There are several limitations to our study. Firstly, the study included only eight female patients, which limits the generalizability of the findings and may affect the statistical power; however, it is the first to propose an automatic method that can be performed retrospectively on CT without requiring contrast agents or a special acquisition sequence. Further research with larger cohorts is essential to validate this method comprehensively and to ascertain the specific type of fibrosis (focal vs. diffuse) that this parameter may represent in the future. The study included only female patients with HER2-positive breast cancer, which may limit the applicability of the findings to other populations, including males or patients with different cancer types. Another important limitation of the study is the absence of correlation with histopathological findings, which is why it cannot be known if the percentage calculated in the 60–90 HU range is exclusively caused by fibrosis and not by another associated process, such as microvascular ischemia. The study relies on specific imaging modalities (CT and CMR) for the detection of myocardial fibrosis. Variability in imaging quality or protocols may impact the results. The identification of LGE plays an important prognostic role, both in ischemic and non-ischemic cardiomyopathies, correlating with an increased odds ratio for sudden cardiac death [34]. Given the observed correlation between the fibrosis percentage determined by the algorithm and LGE, it would be valuable to assess in future studies the extent to which fibrosis identification on native CT sequences can provide similar prognostic information. However, the study aimed to offer preliminary data needed for prospective sample size calculations. For an alpha error of 0.05, using a beta error threshold of 0.20, and based on fibrosis percentages obtained for the patients in this study, we calculated that a minimum of 62 patients would need to be enrolled for a final validation of our algorithm. Given the observed correlation between the fibrosis percentage determined by the algorithm and LGE, we strongly feel such a final validation would be a valuable assessment by offering fibrosis identification on native CT sequences with similar prognostic information.

## 5. Conclusions

The present pilot study demonstrates encouraging results for the newly developed, automatic, computer-assisted method that proposes a novel imaging marker for identifying and quantifying myocardial fibrosis. A significant advantage of this innovative approach is that it extracts data from native CT images, making it a faster and more cost-effective alternative to CMR. The method was tested on a small cohort of HER2-positive breast cancer patients, evaluated using both CT and CMR, and exhibited optimal reliability along with high to very high correlations with CMR-derived fibrosis parameters.

## Figures and Tables

**Table 1:** Summary of the clinical characteristics.

Clinical Characteristics	Patients (n = 8)
Age	54.88 ± 12.05
Weight (kg)	87.38 ± 15.14
Height (cm)	164.38 ± 7.98
BMI (kg/m^2^)	32.21 ± 4.3
Pulse rate (bpm)	72.88 ± 10.93
SBP (mmHg)	133.75 ± 13.02
DBP (mmHg)	78.75 ± 13.82
Total cholesterol (mg/dL)	203 ± 46.91
HDL-C (mg/dL)	45.13 ± 8.43
LDL-C (mg/dl)	125.38 ± 37.97
Tg (mg/dL)	161.75 ± 71.21
Blood glucose (mmol/L)	113.38 ± 11.87
Creatinine (mg/dL)	0.76 ± 0.18
GFR (ml/min/1.73 m^2^)	87.88 ± 22.15
Hypertension (%)	50
Diabetes (%)	12.5
Smoker status	
Active (%)	12.5
Non-smoker (%)	75
Ex-smoker (%)	12.5
Symptoms	
Dyspnea (%)	75
Chest pain (%)	25
Palpitations (%)	37.5
ECG findings	
Non-specific ST-T changes (%)	62.5
Medication	
Beta blocker (%)	87.5
ACE inhibitors (%)	75
Diuretics (%)	62.5
Calcium blocker (%)	0
Statins (%)	50
Antiplatelets drug (%)	25
NT-proBNP, (IQR) pg/mL	785 (290–960)

BMI body mass index; SBP systolic blood pressure; DBP diastolic blood pressure; PP: pulse pressure; Tg triglycerides; GFR glomerular filtration rate; ECG electrocardiogram; ACE angiotensin-converting enzyme; The results are reported as mean ± SD, if not otherwise specified.

**Table 2:** CMR and CCT characteristics of the enrolled patients.

	CMR (n = 8)	CT (n = 8)	*p*
LVEDVI, mean (SD), mL/m^2^	65.0 (11.6)	60.4 (13.1)	0.07
LVESVI, mean (SD), mL/m^2^	35.0 (6.4)	30.9 (8.4)	0.076
LVEF, mean (SD), %	46.3 (3.7)	48.8 (1.1)	0.579
LV mass, g/m^2^ (SD)	71.8 (11.2)	64 (15.3)	0.027
GLS, mean (SD), %	−12.25 (3.87)	-	NA
LGE+, n (%)	7 (87.2)	-	NA
Myocardial localization—septal/lateral/anterior/inferior/circumferential	3/1/2/2/0	-	NA
Myocardial pattern—subepicardial/nodal/midwall	2/5/1	-	NA
LV-LGE, mean (SD), g	24.8 (12.3)	-	NA
LV-LGE/LV mass, %	17.9 (8.9)	-	NA
Native T1 mapping, mean (SD), ms	1114 +/−51	-	NA
ECV, mean (SD), %	31.2 (3.1)	-	NA
Pericardial effusion+, n (%)	3 (37.5)	-	NA

LVEDVI left ventricle end-diastolic volume index; LVESVI left ventricle end-systolic volume index; LVEF left ventricle ejection fraction; LV left ventricle; GLS global longitudinal strain; LGE late gadolinium enhancement; ECV extracellular volume.

**Table 3:** Percentages of fibrosis generated by the algorithm when different CT images of the same patient are chosen.

Fibrosis Percentage as Returned by the Algorithm (%) with 3 Different Images of the Same Patient						
Patient	#1	#2	#3	Mean	SD	CV (%)
1	8.9	9.2	8.79	8.96	0.21	1.93
2	5.77	7.57	6.51	6.62	0.90	11.16
3	7.51	9.75	9.59	8.95	1.25	11.40
4	13.87	11.08	10.52	11.82	1.79	12.39
5	6.53	9.17	9.4	8.37	1.59	15.56
6	11.53	10.4	11.04	10.99	0.57	4.21
7	5.68	4.86	5.73	5.42	0.49	7.35
8	9.71	9.94	8.97	9.54	0.51	4.34

CV coefficient of variance; # number of image.

**Table 4:** Fibrosis percentage, as reported by the algorithm, after running it 3 times by the same observer, for the region of interest identified using a different number of iterations for the Snake algorithm.

	Fibrosis Percentage as Returned by the Algorithm (%)				
Patient	#1	#2	#3	Mean	CV (%)
1	8.9	9.09	8.82	8.94	1.27
2	5.77	5.81	5.81	5.80	0.33
3	7.51	7.79	7.4	7.57	2.17
4	13.87	13.85	13.64	13.79	0.75
5	6.53	6.78	6.53	6.61	1.78
6	11.53	11.55	11.53	11.54	0.08
7	5.68	5.11	5.75	5.51	5.20
8	9.71	9.83	9.71	9.75	0.58

CV coefficient of variance; # number of measurement.

## Data Availability

The data presented in this study are available on request from the corresponding author due to privacy constraints.
